# Undernutrition and associated factors among urban children aged 24–59 months in Northwest Ethiopia: a community based cross sectional study

**DOI:** 10.1186/s12887-019-1595-3

**Published:** 2019-06-29

**Authors:** Aweke Girma, Haile Woldie, Fantahun Ayenew Mekonnen, Kedir Abdela Gonete, Mekonnen Sisay

**Affiliations:** 1 Department of Human Nutrition, Institute of Public Health, College of Medicine and Health Sciences, University of Gondar, Gondar, Ethiopia; 2 Department of Epidemiology and Biostatistics, Institute of Public Health, College Medicine and Health Sciences, University of Gondar, Gondar, Ethiopia

**Keywords:** Preschool children, Undernutrition, Stunting, Wasting, Underweight, Northwest, Ethiopia

## Abstract

**Background:**

Globally, in every three preschool children one is affected by malnutrition. In Ethiopia, child undernutrition continues to be a serious public health problem. Data are scarce, especially in 24-59 months age children. We aimed at estimating under nutrition and its associated factors among children 24–59 months age in Aykel Town, Northwest Ethiopia.

**Methods:**

A community based cross-sectional study was conducted among children aged 24–59 months in Aykel Town from January to February 2017. A total of 416 children were included in to the study using a systematic random sampling technique. Data were collected by interview and anthropometric measurements. Multivariable analysis was performed to identify the predictors of stunting, wasting and underweight.

**Results:**

The prevalence of stunting, wasting and underweight were 28.4, 10 and 13.5%, respectively. Children from low birth order; 1^st^ (AOR = 8.60, 95%CI: 2.40, 3.70) and 2nd -4th (AOR = 5.80, 95%CI: 1.80, 18.90), from large family size (AOR = 3.67, 95%CI: 1.92, 7.00), and had meal frequency < 3/day (AOR = 5.09, 95%CI: 2.96, 8.74) were at a higher risk of stunting. Children who had not fed on cow milk (AOR = 5.50, 95%CI: 2.30, 13.00), and from mothers who had poor hand washing practice (AOR = 11.00, 95%CI: 4.30, 27.9) were more likely to be wasted. Children who had not fed on cow milk (AOR = 2.90, 95%CI: 1.40, 6.00), breast fed for less than 24 months (AOR = 2.60, 95%CI: 1.35, 5.00), consumed foods from less than four food groups (AOR = 6.30, 95%CI: 1.70, 23.00), and were from mothers’ who had poor hand washing practice (AOR = 2.50, 95%CI: 1.30, 4.70) had higher odds of being underweight.

**Conclusion:**

Stunting, wasting and underweight are high among children aged 24–59 months in Aykel Town. Poor child feeding and maternal hygienic practices were identified as risk factors of undernutrition. Educating mothers/care givers on the advantages of proper child feeding and maintaining hygienic practices at critical times is valuable in improving the nutritional status of children.

## Background

Undernutrition among children continues to be a major public health problem throughout the world. Globally, one in every three under-five children is undernourished. In 2018, about 155 million children under the age of 5 years were stunted and 52 million wasted worldwide. Asia and Africa contributed 56 and 38% of stunting, and 69 and 27.2% of wasting, respectively, of the global undernutrition burden [1].

Wasting is a measure of acute undernutrition, and it may result from inadequate food intake or from a recent episode of illness that caused weight loss. Stunting is a sign of chronic undernutrition that reflects failure to receive adequate nutrition over a long period and can also be affected by recurrent and chronic illness [2].

Among all under five children deaths, childhood malnutrition is responsible for 35% of deaths. More than 2 million children die each year as a result of undernutrition before the age of 5 years [3].

Undernutrition has a negative impact on cognitive development, school performance and productivity [4, 5]. In addition to this, childhood undernutrition poses low adult wages, overweight, obesity, and nutrition-related chronic diseases during adolescent and adulthood periods [4, 6].

Even though the prevalence of stunting is reduced in Ethiopia currently, the burden of undernutrition has still remained as a severe public health problem in spite of the fact that the country has implemented a comprehensive nutritional intervention programs over the past decades [7, 8]. Of the under five children, about 38% were stunted, 10% wasted and 24% underweight in 2016. Concerning the trends of undernutrition among children aged 24–59 months in 2011 and 2016, stunting had reduced from 54 to 46%, and underweight reduced from 32.7 to 27%, while wasting remained unchanged [2, 9].

Undernutrition in children occurs due to the interplay of several factors, which include variables related to the maternal age, maternal education, poor feeding practice, maternal nutritional status, parity and multiple births, sex of the child, illness, birth interval and immunization status, poor wealth status, large families, water and sanitation, place of residence, and other factors relating to health services utilization [10–13].

Majority of the studies conducted in Ethiopia and abroad have focused on children aged between 6 and 23 and 6–59 months. Children aged between 24 and 59 months have rapid growth and development, and is a period when nutrient requirement is highest, and there is a change in their dietary habits. However, data are scarce in this age groups. Therefore, this study was aimed to bridge the knowledge gap on comprehensive understanding of magnitude and determinants of all forms of undernutrition (stunting, wasting and underweight) and their predictors among children aged between 24 and 59 months in Aykel Town, Northwest Ethiopia.

## Methods

### Study design and setting

A community based cross-sectional study was conducted among children aged between 24 and 59 months in Aykel Town from January to February 2017. Aykel Town is located in Chilga district, Northwest Ethiopia about 780 km away from the capital city of Ethiopia, Addis Ababa. There are two kebeles (the smallest administrative unit in Ethiopia) in the Town and each kebele has four villages. The Town has one hospital, one health center and two urban health posts. It has a total of 4246 households and total population of 30,201 among which 16,498 (54.6%) are females. Of the total population, 1768 (6%) are preschool children. The people in this area are engaged in different activities such as farming, trade, civil servant, carpentering, and construction. They also produce cereals, legumes and spices and root crops, and keep animals, including goats, sheep and cattle [14].

### Study participants

All children aged 24–59 months with their mothers/caregivers in Aykel Town were the study population. All randomly selected children aged 24–59 months with their mothers/caregivers who lived in the Town for at least 6 months were included in to the study. Children who were seriously ill, with diarrhoea and /or malaria, and whose mothers’/care givers were unable to communicate were excluded from the study.

### Sample size and sampling procedure

The minimum sample size was determined by using single population proportion formula. The prevalence of undernutrition in the specific age group (24–59 months) was used to calculate the sample size. The prevalence of stunting, wasting and underweight were 57, 16 and 25%, respectively [15]. Considering 95% confidence interval and 5% margin of error, the largest sample size was taken after sample size was calculated for the three indicators of under nutrition. The prevalence of stunting provided the largest sample size. Finally, a sample size of 416 was obtained after considering a 10% non-response rate.

Regarding the sampling procedure, both kebeles of the Aykel Town were included in the study. There were 4246 households in the two kebeles. The total number of households (1768) with children aged 24–59 months was obtained from the Health Extension Workers housing registration. Then households were sampled from each kebele based on proportional allocation. Finally, a systematic random sampling technique was used to select households with eligible children. The first household was selected by lottery method from the first four households by spinning a pen at the center of the Town. Where impossible to get preschool aged children, the next house was considered for the study. When there were more two or more children in the household, one of them was selected by lottery method.

### Data collection tools and procedures

Interviewer-administered questionnaire was used to collect data on socio-demographic and other relevant child and mothers/caregivers related information. To maintain its consistency, the questionnaire was first prepared in English and translated into Amharic, the local language of the study area, and then back translated to English. Six data collectors and two supervisors were involved in the data collection process. Two days training has been given for both data collectors and supervisors on areas related to anthropometric measurements and interview techniques. The questionnaire was pre-tested on 5% of the total sample size outside of Aykel Town. Based on the results of the pre-test, the acceptability and applicability of the procedures and tools were assessed. Necessary revisions were made on the questionnaire.

### Operational definitions and measurements

**Early initiation of breastfeeding:** Children who received breast milk within 1 h of birth [16]

**Exclusive breastfeeding:** Children who received breast milk exclusively up to 6^th^ months of life [16].

**Continued breastfeeding at 1 year:** Children 12–15 months of age who continued breast feeding after the age 1 year [16]

**Introduction of solid, semi-solid or soft foods:** Children who received solid, semi-solid or soft foods during the age of 6–8 months [16]

**Minimum dietary diversity:** Children 6–23 months of age who received foods from 4 or more food groups in the past 24 h [16]

**Minimum meal frequency:** Children from 6 to 23 months of age who received solid, semi-solid, or soft foods (including milk feeds for non-breastfed children) for at least three times per day in the past 24 h [16]

**Continued breastfeeding at 2 years:** Children 20–23 months of age who continued breast feeding after his/her 23 months of age [16]

**Duration of breastfeeding:** Median duration of breastfeeding among children less than 36 months of age [16]

**Under-nutrition:** Refers to a state/condition/ resulting from deficiency of one or more essential nutrients and manifested by stunting, wasting and underweight [17]

**Stunting: Wasting: Underweight:** Refer to a low height for age, weight for height and weight for age, respectively. The child was classified as stunted, wasted and underweight if his/her z score was less than −2SD; otherwise, he/she was considered as well-nourished (≥ − 2 Z score), based on international median of WHO reference value, taking sex into consideration [17].

Weight was measured with light cloths and no shoes by using beam balance in kilogram to the nearest of 0.1 kg. A vertical measuring board was used to measure the height of children. The child stands up on the board barefooted; have hands putting loosely with feet parallel to the body, and heels, buttocks, shoulders calve and back of the head touching the board. Child’s head was held straight comfortably with the lower border of the orbit of the eye being in the same horizontal plane as the external canal of the ear. The head piece of the measuring board was then moved gently, touching the hair and making contact with the top of the head. Height was read to the nearest 0.1 cm [17].

To determine the minimum dietary diversity score (DDS) of the child, the mother was asked to list all food items consumed by the child in the previous 24 h ahead of data collection. Then, the listed food items were grouped in to seven food groups. Namely grains, roots and tubers, legumes and nuts; Dairy products (milk, yogurt, cheese); flesh foods (meat, fish, poultry and liver/organ meats); Eggs, Vitamin-A rich fruits and vegetables and Other fruits and vegetables. Considering the four food groups as the minimum acceptable dietary diversity, a child with a DDS of less than four was classified as poor dietary diversity [18].

Initiation of complementary feeding was measured as early initiation, timely initiation and lately initiation if the mother initiated complementary feeding to the index child before sixth month, at sixth month and after sixth months of age, respectively [16].

Appropriate hand washing practice is defined through 2 questions. The first question was as to when do mothers/caregivers wash their hands. The possible answers for this question were after defecation, after cleaning baby’s bottom, before food preparation, before eating, before feeding children (including breastfeeding). The second question was about how the mothers/caregivers wash their hands. The possible answers for this question were uses water, uses soap or ashes, washes both hands, rubs hands together at least three times, and dries hands hygienically by air-drying or using a clean cloth. A score of 9 points or more (out of a possible 10 points) qualifies a hand washing behavior as appropriate [19]. Wealth index of the household was constructed using household assets data via a principal component analysis to categorize the household wealth index in to lowest, middle, and highest.

Household food insecurity was measured using Household Food Insecurity Access Scale (HFIAS) measurement tools of FAO-FANTA. The HFIAS consists of 9 items specific to an experience of food insecurity occurring within the previous 4 weeks. Respondents were asked whether they had encountered the items because of lack of food or money to buy food in the last month. Each item was received either 1 for occurrence or 0 for non-occurrence. The frequency scores were ranged from 0 to 3, while 0 was the score for non-occurrence, 1 for rarely (once or twice in the past 4 week), 2 for sometimes (three to ten times in the past 4 weeks), and 3 for often (more than ten times in the past month), renamed as food secure, mildly food insecure, moderately food insecure, and severely food insecure, respectively [20].

### Data quality control

Maximum efforts were made to maintain the quality of data. The measuring equipment was calibrated using a known weight material each day. At the end of every data collection day, each questionnaire was examined for its completeness and consistency by field supervisors and investigators and pertinent feedback was given to data collectors and supervisors.

### Data processing and analysis

Data was coded, cleaned, and entered in to Epi-Info version 3.5.3 and then exported to Statistical Package for Social Sciences (SPSS) version 20 for further analysis. Anthropometric measurements were converted into Z-sore values using WHO Anthro version 3.2.2 software for the indices; Height for- Age (HAZ), Weight-for-Height (WHZ) and Weight-for- Age (WAZ) taking sex into consideration using WHO 2006 standards. The child was classified as stunted, wasted and underweight if his/her z score was less than −2SD; otherwise, he/she was well-nourished (≥ − 2 Z score) [17]. Mean with standard deviation for continuous and proportion for categorical variables were calculated. Binary logistic regression model was used to identify factors associated with stunting, wasting and underweight. Variables which had a *p*-value of < 0.2 in the bivariable analysis were taken in to the multivariable analysis to control the possible effects of confounders. Adjusted odds ratio (AOR) with a 95% confidence interval (CI) was computed to assess the strength of the association. A *p*-value of < 0.05 was used to determine statistical significance in the multivariable analysis. The Hosmer and lemeshows goodness of fit-test was run to check the fitness of the final model for the three separate models.

### Ethical considerations

Ethical clearance was obtained from Ethical Review Board of University of Gondar. An official permission letter was secured from Aykel Town administration health office. Participants were involved in the study on a voluntary basis after written consent, signed or verified by fingerprint was obtained. Parents/care givers of the children were informed about the study and written consent on behalf of the children were obtained from the parents. Privacy of the participants was protected and the information was kept confidential.

## Results

### Socio- economic and demographic characteristics of study participants

A total of 401 child-mother pairs were participated in the study, making a response rate of 96.4%. The mean (±SD) age of the children was 41.24 (±10.64) months, and the sex ratio of participants was almost equal (50.4, 49.6%). The majority of the mothers/care givers were married, 371(92.5%), and Orthodox Christians, 351(87.5%). More than half, 244 (60.3%), of the mother/care givers were housewives. Nearly one-third, 129 (32.2%), of the households had > 4 family size. Over three-fourth of the mothers, 324 (80.8%), and fathers, 305 (76%), were at least at their primary level of education (Table 1).

**Table 1:** Socio- economic and demographic characteristics of study participants in Aykel Town, Northwest Ethiopia, 2017

Variables	Frequency (%)
Child age (month)	
24–35	155 (38.7)
36–47	131 (32.7)
48–59	115 (28.7)
Sex ratio	1.02:1
Marital status	
Married	371 (92.5)
Single	3 (0.7)
Divorced	25 (6.2)
Widowed	2 (0.5)
Religion	
Orthodox	351 (87.5)
Muslim	50 (12.5)
Number of children	
≤ 2	311 (77.6)
3–5	73 (18.2)
≥ 6	17 (4.2)
Birth order of a child	
1^st^	211 (52.6)
2^nd^ -4^th^	158 (39.4)
≥ 5^th^	32 (8.0)
Maternal education	
Unable to read and write	73 (18.2)
Able to read and write	4 (1.0)
Primary education	58 (14.5)
Secondary education	132 (32.9)
Diploma and above	134 (33.4)
Maternal occupation	
House wife	244 (60.3)
Civil servant	126 (31.4)
Merchant	28 (7.0)
Daily labourer	3 (0.3)
Paternal education	
Unable to read and write	88 (21.9)
Able to read and write	8 (2.0)
Primary education	64 (16.0)
Secondary education	108 (26.9)
Diploma and above	133 (33.2)
Paternal occupation	
Farmer	102 (25.3)
Civil servant	157 (39.2)
Merchant	91 (22.7)
private employ	21 (5.3)
Daily labourer	30 (7.5)
Household size/family size	
≤ 4	272 (67.8)
> 4	129 (32.2)
Wealth index	
Lowest	161 (40.1)
Middle	79 (19.7)
Highest	161 (40.2)
Household food security	
Food secure	276 (68.8)
Mildly food insecure	106 (26.4)
Moderately food insecure	19 (4.8)

### Health and nutrition related characteristics of study participants

Almost all, 410 (99.8%), of the children were fully immunized. Eighty percent of the children were born at the health facility. Over three-fourth of the children, 323 (80.5%), had dietary diversity score less than four food groups. Majority, 354 (88.3%), of the mothers initiated breast feeding timely. Most of mothers didn’t practice pre-lacteal feeding, 341 (85%), but started complementary feeding at the 6^th^ month of age, 326 (81.3%) (Table 2).

**Table 2:** Health and nutrition related characteristics of study participants in Aykel Town, Northwest Ethiopia, 2017

Variables	Frequency (%)
Place of delivery	
Home	79 (19.7)
Health facility	322 (80.3)
Initiation of breast feeding	
Early	354 (88.3)
Late	47 (11.7)
Pre-lacteal feeding	
Yes	60 (15.0)
No	341 (85.0)
Initiation of complementary food	
Timely	326 (81.3)
Not Timely	75 (18.7)
Dietary diversity score	
> 4 food groups	78 (19.5)
< 4 food groups	323 (80.5)
Meal frequency	
< 3/day	118 (29.4)
≥ 3/day	283 (70.6)

### Hygiene and environmental sanitation related characteristics

Regarding household water source for domestic consumption, almost all, 398 (99.3%), of the participants reported that they used from protected source and were able to access water within 30 min walking distance. Most of the households had latrine, 397 (97.3%), and more than half of the respondents had good hand washing practice, 248 (61.8%) (Table 3).

**Table 3:** Hygiene and environmental sanitation related characteristics of study participants in Aykel Town, Northwest Ethiopia, 2017

Variables	Frequency (%)
Source of drinking water	
Protected	398 (99.3)
Unprotected	3 (0.3)
Availability of latrine	
Yes	390 (97.3)
No	11 (2.7)
Utilization of latrine	
Yes	390 (97.3)
No	11 (2.7)
Hand washing practice	
Good	248 (61.8)
Poor	153 (38.2)
Solid waste management practice	
Good	332 (82.8)
Not good	69 (17.2)
Availability of liquid waste disposal pit	
Yes	107 (26.7)
No	294 (73.3)

### Prevalence of undernutrition

Of the total children participated in the study, 28.4% were stunted, 10% wasted and 13.4% underweight. The prevalence of the mixed undernutrition was found to be 0.25% for both stunting and wasting, 8.23% for stunning and underweight, and 4.99% wasting and underweight.

#### Factors associated with undernutrition

Independent variables believed to result in undernutrition, according to previous evidences and experiences, were examined for possible associations with stunting, wasting and underweight using a binary logistic regression model. A bivariable analysis was first performed to identify predictor variables associated with the outcome variables. The factors having a *p*-value of < 0.2 were in the bivariable model were taken to the multivariable model to see their independent association with stunting, wasting and underweight.

#### Factors associated with stunting

In the multivariable analysis, birth order, meal frequency and family size were significantly associated with stunting. Being the first child in the birth order increases the odds of developing stunting by 8.6 times (AOR = 8.60; 95% CI: 2.40, 30.77). The probability of being stunted was 5.8 times higher among children whose birth order was 2^nd^ -4^th^ (AOR = 5.80, 95%CI: 1.80, 18.90). Meal frequency of less than three times per day (AOR = 5.10, 95%CI: 2.96, 8.74) and large family size (AOR = 3.67, 95%CI: 1.92, 7.00) increase the odds of being stunted (Table 4).

**Table 4:** Factors associated with stunting among children aged 24–59 months in Aykel town, Northwest Ethiopia, 2017

Variables	Stunted		COR (95%CI)	AOR (95%CI)
	Yes	No		
Maternal education				
Unable to read and write	24	49	2.07 (0.86,4.96)	1.91 (0.51,7.18)
Read and write only	1	3	1.40 (0.13,15.20)	2.82 (0.04,189.00)
Primary education	25	33	3.20 (1.30,7.81)	2.55 (0.69,9.47)
Secondary education	36	96	1.58 (69.00,3.60)	1.77 (0.56,5.66)
Diploma and above	28	106	1	1
Paternal education				
Unable to read and write	33	55	2.26 (1.14,4.49)	1.92 (0.55,6.76)
Read and write only	0	8	0.00 (0.00,-)	0.00 (0.00, −)
Primary education	24	40	2.26 (1.08,4.70)	1.36 (0.41,4.59)
Secondary education	31	77	1.52 (0.77,2.99)	1.17 (0.43,3.21)
Diploma and above	26	107	1	1
Child age (month)				
24–35	40	115	1.08 (0.62,1.85)	1.09 (0.57,2.07)
36–47	46	85	1.68 (0.98,2.90)	1.33 (0.69,2.55)
48–60	28	87	1	1
Birth order				
1^st^	56	155	1.56 (0.61,4.00)	8.60 (2.4, 3.70)**
2^nd^ -4^th^	52	106	2.13 (0.82,5.48)	5.80 (1.8,18.90)**
≥ 5^th^	6	26	1	1
Adequacy of water				
Adequate	23	82	0.63 (0.37,1.06)	0.81 (0.42,1.56)
Inadequate	91	205	1	1
Meal frequency				
< 3/day	61	57	4.64 (2.90,7.42)	5.09 (2.96, 8.74)***
≥ 3/day	53	230	1	1
Family size				
≤ 4	60	212	0.39 (0.25,0.62)	0.27 (0.14,0.54)***
> 4	54	75	1	1
Hand washing practice				
Not good	50	103	1.39 (0.89,2.17)	1.40 (0.83, 2.32)
Good	64	184	1	1
Paternal occupation				
Farmer	37	55	1.57 (0.38,6.46)	1.33 (0.26,6.73)
Civil servant	35	122	0.67 (0.16,2.72)	0.85 (0.14,5.16)
Merchant	24	67	0.84 (0.20,3.49)	1.00 (0.18,5.44)
Private employee	8	13	1.44 (0.29,7.20)	1.13 (0.16,8.23)
Daily labourers	7	23	0.71 (0.14,3.50)	0.75 (0.12,4.78)
Hand craft	3	7	1	1

** Statistically significant variables at *p* < 0.01, *** statistically significant variables at *p* < 0.001, the result of Hosmer and Lemshow test was > 0.24

#### Factors associated with wasting

The multivariable logistic regression analysis showed that cow milk feeding of children and hand washing practice of mothers’/care takers’ were significantly associated with wasting. An increased odds of wasting were observed among children who were not fed on cow milk (AOR = 5.48, 95%CI: 2.29, 13.09), and whose mothers had poor hand washing practice (AOR = 11.00, 95%CI: 4.34, 27.90) (Table 5).

**Table 5:** Factors associated with wasting among children aged 24–59 months in Aykel town, Northwest Ethiopia, 2017

Variables	Wasted		COR(95%CI)	AOR (95%CI)
	Yes	No		
Paternal education				
Unable to read and write	8	80	1.06 (0.36,3.06)	0.80 (0.23,2.95)
Read and write only	4	4	10.57 (2.16,51.70)	5.98 (0.92,38.86)
Primary education	7	57	1.30 (0.43,3.90)	0.56 (0.14,2.18)
Secondary education	10	98	1.08 (0.40,2.97)	1.13 (0.36,3.56)
Diploma and above	11	122	1	1
Child age in month				
24–35	19	136	1.09 (0.51, 2.30)	1.11 (0.46,2.67)
36–47	8	123	0.51 (0.20,1.28)	0.52 (0.18,1.46)
48–60	13	102	1	1
Number of children				
1–2	27	284	0.31 (0.09,1.0)	0.76 (0.10,5.71)
3–5	9	64	0.46 (0.12,1.71)	0.94 (0.13,6.86)
6–10	4	13	1	1
Birth order				
1^st^	18	193	0.33 (0.13,0.87)	0.41 (0.07,2.27)
2^nd^ -4^th^	15	143	0.37 (0.14,1.01)	0.37 (0.07,1.92)
≥ 5^th^	7	25	1	1
Wealth index				
Poor	11	150	0.60 (0.30,1.50)	0.60 (0.23,1.56)
Medium	13	66	1.78 (0.80,3.90)	2.26 (0.89,5.72)
Rich	16	145	1	1
Hand washing practice				
Not good	33	120	9.50 (4.07, 22)	11.00 (4.34,27.90)***
Good	7	241	1	1
Milk feeding				
Yes	24	305	0.27 (0.14,0.55)	0.18 (0.08,0.44)***
No	16	56	1	1

*** Statistically significant variables at *p* < 0.001, the result of Hosmer and Lemshow test was > 0.47

#### Factors associated with underweight

Duration of breast feeding, dietary diversity score, lack of cow milk feeding and hand washing practice of mothers were significantly associated with underweight. The likelihood of being underweight was 2.6 times higher among children who fed on breast for < 24 months compared to those who fed on breast for 24 and more months (AOR = 2.6, 95%CI: 1.35, 5.00). Lower dietary diversity score (AOR = 6.33, 95%CI: 1.73, 23.1) and poor hand washing practice of mothers were found to result in an increase in the odds of being underweight (AOR = 2.50, 95%CI: 1.3, 4.7) (Table 6).

**Table 6:** Factors associated with underweight among children aged 24–59 months in Aykel town, Northwest Ethiopia, 2017

Variables	Underweight		COR (95%CI)	AOR (95%CI)
	Yes	No		
Maternal education				
Unable to read and write	8	65	1.81 (0.45,7.18)	1.20 (0.27, 5.33)
Read and write only	2	2	14.67 (1.50,144)	15.00 (1.32, 17.10)
Primary education	12	46	3.83 (1.00,14.5)	2.10 (0.50,8.80)
Secondary education	15	117	1.88 (0.52,6.80)	1.50 (0.37,5.78)
Diploma and above	17	117	1	1
HHs Food security				
Food secure	32	244	0.28 (0.10,0.80)	0.26 (0.08,0.82)*
Mild food insecure	16	90	0.38 (0.13,1.16)	0.35 (0.11, 1.36)
Moderately food insecure	6	13	1	1
Duration of breast feeding				
Adequate	29	250	0.45 (0.25,0.80)	0.38 (0.20, 0.74)**
Inadequate	25	97	1	1
Dietary diversity score				
≥ 4 food groups	3	75	0.21 (0.06,0.70)	0.16 (0.04, 0.57)**
< 4 food groups	51	272	1	1
Hand washing practice				
Not good	31	122	2.48 (1.39,4.45)	2.50 (1.30,4.70)**
Good	23	225	1	1
Cow Milk feeding				
Yes	38	291	0.46 (0.24,0.87)	0.35 (0.17,0.72)**
No	16	56	1	1
Meal frequency				
< 3/day	17	101	1.12 (0.60,2.08)	1.10 (0.55,2.20)
≥ 3/day	37	246	1	1

* Statistically significant variables at *p* < 0.05, ** statistically significant variables at *p* < 0.01, the result of Hosmer and Lemshow test was> 0.15

## Discussion

The prevalence of stunting, wasting and underweight were 28, 10 and 13.4%, respectively, among children aged 24–59 months.

The prevalence of stunting and underweight reported in this study was congruent with a study done in Bure Town, Ethiopia, which showed a prevalence of stunting to be 24.9% and underweight 14.3% [21]. This may be due to contextual similarities like socio- demographic and economic characteristics. However, these prevalence rates were lower than the study findings of Ethiopian Demographic and Health Survey 2016 [22], a study conducted in Lalibela Town, Ethiopia [23], and Somalia region, Ethiopia [15], which reported higher prevalence of stunting and underweight among under five children. The reason for the differences with the EDHS 2016 survey finding may be the fact that the EDHS survey was a nationwide study on large sample size, encompassed urban and rural areas, while the current study was conducted on urban population only. The lower prevalence in the current study as compared to the findings in Lalibela and Somalia region could be because these areas are among the repeatedly drought affected areas.

Compared to the WHO cut-off values for public health significance of undernutrition, the prevalence of wasting (10.0%) remained as a serious public health problem in the study area [24]. The finding was in line with reports of other studies in Ethiopia, such as from Ethiopian Demographic and Health Survey report (9%) [22] and a study in Lalibela Town (8.9%) [23]. However, compared to this study, higher prevalence of wasting was reported from the pastoralist area of Somali region, Ethiopia (42.3%) [15]. This might be because Somali is drought affected region. In general, the findings of this study revealed that undernutrition is a serious public health problem in the study area among under five children which indicated that nutrition specific, nutrition sensitive and other modalities of intervention programs should be strengthen and strongly implemented to reduce the magnitude of undernutrition and its consequences.

This study revealed that birth order of the child was significantly associated with stunting. Stunting decreases as birth order increases. This finding was in line with other study findings in Ethiopia and abroad. For instance, a study done in Ethiopia revealed that children of lower birth order are at higher risk of developing stunting than higher birth order [25]. Another study in Bangladesh also showed that children born later in the birth order have an additional advantage [26]. This can be explained due to the fact that mothers may have an experience in terms of place and method of delivery, awareness about importance of antenatal and postnatal follow up and optimal breast feeding if they had prior birth experience. The odds of stunting among children who had low meal frequency (less than 3 times per day) were high. This result was supported by studies done in Afghanistan [27], which showed that significantly higher proportion of stunted children were found among those who were fed less than 3 times per day. This study result should that due attention should be given to all children in the household in terms of feeding, caring and responsiveness, age appropriate feeding and adequate meal frequency by parents.

This study revealed that children who did not fed on cow milk were more likely to be wasted and underweight as compared to those who fed on cow milk as a complementary food. This result is in line with a study done in the United States [28]. This might be because animal source foods are a richer source of both macro and micronutrients [29]. In addition, cow milk is more concentrated and denser than human milk in its nutrient contents; such as protein, fat and calcium which are among the most important nutrients needed for child growth and development. This implied that children should be introduced with appropriate complementary foods at the age of 6 months. At the time of introducing complementary feeding, children usually preferred liquid and semisolid foods than solid foods. Therefore, besides other complementary foods cow milk is an important source of nutrients and should be given for children with due safety.

Furthermore, the odds of wasting and underweight were higher among children whose mothers’/caretakers’ hand washing practice was poor as compared to those children whose mothers’/caretakers’ had good hand washing practice. This finding was supported by study findings reported from Ethiopia [30] and Uganda [31] which revealed that wasting and underweight were associated with improper personal hygiene. This is because of washing hands at critical times prevent various communicable disease. Hand washing with soap before feeding children and after cleaning them can interrupt the transmission of feco-oral microbes in the domestic environment [32]. This finding showed due concern should be given for improved hand washing and hygiene in order to prevent diarrheal diseases and other infections.

The current study indicated that children with low dietary diversity score were at higher risk of developing underweight in comparison with those children who consumed food from four and more food groups in the past 24 h prior to the survey. The odds of underweight among children with low dietary diversity scores (< 4) were 6.3 times higher as compared to children with better dietary diversity scores (≥4). A study done in Somali region, Ethiopia showed that low dietary diversity score was a significant predictor of underweight [32]. Another study done in Sidama Zone, Ethiopia reported that dietary inadequacy and low diet quality in terms of diversified diet and availability of micronutrients had a significant negative association with child growth [33]. Dietary diversity is a proxy measure of an individual’s diet quality. Therefore, as the result indicates, nutrition of children should get due attention concerning consumption of balanced/diversified foods.

The other factor associated with underweight was duration of continued breastfeeding. The odds of underweight among children who discontinued breast feeding before the age of 24 months was 2.6 times higher as compared to those children who breast fed beyond 24 months. This finding is similar with a findings of a study done in South Africa [34]. This might be due to the association between breast feeding and weight gain [35], and the association between breastfeeding and lower incidence of childhood infection. This might also be due to the immunological advantage of breast feeding. Since breast milk has a balanced nutrient in it, mothers should be encouraged to continue breast feed their children up to 2 years and beyond.

### Limitations of the study

This study has attempted to determine the magnitude and factors associated with undernutrition (stunting, wasting and underweight) among children aged 6–59 months in the urban community (Aykel Town), Northwest Ethiopia. However, it is without limitations. Firstly, the study did not include some information, like deworming, vitamin supplementation status, and nutritional status of mothers. Secondly, there might be a recall bias in responding for questions related to events happening in the past.

## Conclusion

This study confirmed a high prevalence of undernutrition in Aykel Town. Household family size above 4, being in the birth order of 4th or lower, and hade a meal frequency less 3times per day increase the likelihood of being stunted. The odds of being underweight was higher among children who fed on fewer than 4 food groups (poor dietary diversity score), do not received cow milk as a complimentary food, had lesser duration of breast feeding and whose mothers/care givers had poor hand washing practice. Wasting has significantly affected those children who did not fed on cow milk as a complementary food and whose mother/care givers had poor had washing practice. Proper child feeding, sanitation and hygienic practices need to be enhanced. Education on methods and advantages of limiting family size is curtail in addition.

## Data Availability

Data are available from the corresponding author on reasonable requests.

## Abbreviations

AOR: Adjusted odds ratios
CI: Confidence interval
COR: Crude odds ratio
DDS: Dietary diversity score
EDHS: Ethiopian Demographic and Health Survey
HAZ: Height for- Age
SD: Standard deviation
SPSS: Statistical Package for Social Science
WAZ: Weight-for- Age
WHO: World Health Organization
WHZ: Weight-for- Height
